# Nasal Microbiota and Neuroinflammation: Relationship between Nasal Flora and Multiple Sclerosis Onset/Progression

**DOI:** 10.3390/life12122043

**Published:** 2022-12-07

**Authors:** Federico Maria Gioacchini, Salvatore Ferlito, Massimo Ralli, Alfonso Scarpa, Ignazio La Mantia, Massimo Re, Luigina Romani, Arianna Di Stadio

**Affiliations:** 1 ENT Unit, Department of Clinical and Molecular Sciences, Polytechnic University of Marche, 60121 Ancona, Italy; 2 GF Ingrassia Department, Otolaryngology, University of Catania, 95124 Catania, Italy; 3 Organ of Sense Department, University La Sapienza of Rome, 00185 Roma, Italy; 4 Otolaryngology Department, University of Salerno, 84084 Fisciano, Italy; 5 Microbiology Department, University of Perugia, 06123 Perugia, Italy

**Keywords:** neuroinflammation, nasal microbiota, multiple sclerosis, chronic rhino-sinusal disorder, chronic sinusitis

## Abstract

The role of nasal microbiota in contributing to neuroinflammation is gradually emerging. Multiple sclerosis and chronic rhinosinusitis share important clinical and epidemiological similarities, and the hypothetical connection among these two pathological entities should be carefully investigated. This editorial is based on a review of available literature on this topic. The main international databases were searched using the following keywords: neuroinflammation, nasal microbiota, multiple sclerosis, chronic rhino-sinusal disorders, chronic sinusitis. Four fully-consistent articles that investigated nasal microbiota alteration and/or chronic rhinosinusitis presence in subjects affected by multiple sclerosis were identified. Overall, these studies showed a significant connection between nasal microbiota dysbiosis and the presence of multiple sclerosis. New specific studies to analyze the nasal microbiota and its metabolism in patients affected by multiple sclerosis should be performed. In fact, a series of treatments able to change this flora could improve the rhino-sinusal state with consequent reduction of recurrent episodes of neuro-inflammation.

## 1. Introduction

The role of nasal microbiota has been investigated in neurodegenerative diseases such as Parkinson’s Disease (PD) [1]; however, neither Pereira [2] nor Heintz-Buschart [3] identified any differences in the nasal microbiota composition comparing PD patients and healthy controls. Thangaleela in 2022 speculated that metabolites from nasal microbiota could pass through the Blood Brain Barrier (BBB) [4]. If this hypothesis could be confirmed, the effect of nasal microbiota in the development of neuro-inflammation and neurodegeneration could be like that of gut microbiota [1,5]. Neuro-inflammation is underpinned by the acute, active phenomena of inflammation, so in this phase the composition of nasal microbiota and its change might play an important role in fueling inflammation. The increase of inflammation (and neuroinflammation) could affect the recurrence of Multiple Sclerosis (MS), especially affecting the relapsing phase of the disease. MS is a chronic inflammatory relapsing-remitting disease characterized by demyelination lesions in the central nervous system (CNS). The lesions can cause severe physical or cognitive disability and neurological defects. The origin of the disease could be from a genetic predisposition, environmental factors, or diet [6]. The environment and diet can change the composition of the nasal microbiota [7].

MS can arise due to unknown environmental factors in genetically susceptible individuals. For this reason, several epidemiological studies have been conducted to evaluate occupational risks. An association with farming [8] and exposure to livestock [9] have recently been further narrowed to dairy operators [10]. Inhaled toxins may access the CNS via the nasal mucosa, with the potential to act directly to induce inflammatory neuronal and axonal damage, but they have also the potential to induce nasal microbial dysbiosis with the consequent release of neurotoxic and immune-deviant molecules [11].

Another important trigging factor is Epstein Barr (EBV) infection. The EBV virus, in genetically predisposed subjects, is responsible for “molecular mimicry”. This term indicates the hyper self-immune reactivity that is activated after EBV infection and that is responsible of the neuroinflammation. This increased self-immune response can cause MS [12]. A similar mechanism has been proposed for microbial dysbiosis [12].

The term “microbial dysbiosis” refers to the alteration of healthy microbiota composition, which causes pathological conditions leading to health issues. The microbiota can be both transient and resident. Their diversity is influenced by various factors, such as drugs, surrounding environmental microorganisms, habitat, and nutritional habit. Other host factors can also influence the nasal flora, such as host hygiene, immunity, and genetics, as well as physical factors, such as oxygen, pH, moisture, and other microbial interactions. The colonization of opportunistic pathogens results in the onset of respiratory infections and changes in the innate immune mediators [4].

In animal experiments and clinical research, several studies have suggested that some types of nasal microbiota may enter the brain, contributing to developing neurological diseases [13]. A strong connection between nasal microbiota and CNS neuroinflammation have been already described, in particular for Alzheimer’s disease (AD) and PD [14,15,16,17,18].

This study aims at understanding the link between nasal microbiota, neuro-inflammation and MS based on evidence available in current scientific literature.

## 2. Materials and Methods

Two researchers revised the scientific literature within the platforms PubMed, Scopus, Web of Science and Google Scholar, to identify articles useful to the aim of this paper. The following keywords were used: “neuroinflammation”, “nasal microbiota”, “chronic rhino-sinusal disorders”, “chronic sinusitis”. The keyword “multiple sclerosis” was included in association with each of the previously mentioned keywords. Only articles published in the English language were considered. Papers reporting duplicate data were not included.

The articles reporting potentially relevant information were read in full. The main extracted information was then included in an Excel file (Microsoft Corp, Redmond, Washington, USA) that was separately filled by each researcher. The files were then revised, and concordant results were included in this article.

## 3. Results

Overall, four studies were considered fully consistent with the topic and adherent to the aim of this work. The main features of these studies are reported in Table 1.

## 4. Discussion

### 4.1. The Link between Nasal Microbiota and Neuroinflammation

**Figure 1:**
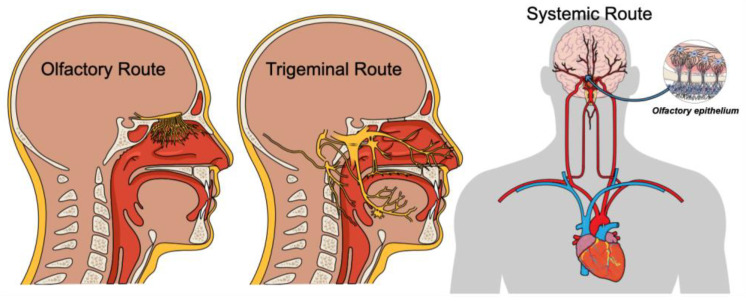
Potential pathways for nasal microbiota to enter the brain. (**Left panel**): the microbiota in the nasal cavity diffuse in the olfactory epithelium via the cribriform plate. (**Central panel**): trigeminal nerve direct route of ascending periaxonal lymphatic flow from its origins in the nose, nasopharynx, maxillary sinus to the brainstem. (**Right panel**): the microbiota can penetrate the rich vascular network in the nasal mucosa, traveling to the brain through the blood brain barrier with the systemic route.

How might nasal microbiota affect brain inflammation? Several studies have been conducted to explain this effect and, to date, three main potential pathways for nasal microbiota to enter the brain have been identified (Figure 1).

(1) *The Olfactory Route*

The nasal microbiota and its products could spread from the nasal cavity up to the olfactory bulbs (OB). The entry route is diffusion through the olfactory epithelium and the cribriform plate up to the OB. Then, from this area the microbiota/ microbiota products spread through the olfactory tubercle in other regions of the brain (basal nucleus, thalamus, hypothalamus, cerebrum, and cerebellum) [23,24]. Several nasal microbiomes, such as HSV-6, bovine herpesvirus type-5, influenza virus, *Streptococcus pneumoniae* and/or SARS-CoV2 (COVID-19), may, via the olfactory bulbs, cause neuroinflammation in the brain [25,26,27,28,29].

(2) *The Trigeminal Route*

The trigeminal pathway is another route to transport microbiota from the nasal cavity to the CNS. The trigeminal nerves (ophthalmic, maxillary, and mandibular), innervates the mucosa of the eyes, noses, oral cavity, and nasopharynx [30]. The nerve trunk conveys its tracers from the mucosa of the maxillary sinus to the brain stem, cerebellum and spinal meninges [31]. Experimentally, by intranasal inoculating of cap mutant strain of *Burkholderia pseudomallei* in the nose of adult mice, it has been shown the bacteria at first invades respiratory epithelium and then colonizes at trigeminal nerves beneath the olfactory epithelium [32]. The immunostaining findings showed that the *Burkholderia pseudomallei* infiltrated the cranial cavity through the trigeminal nerves. These findings are consistent with the theory that the trigeminal nerve provides a direct route of ascending periaxonal lymphatic flow from its origins in the mucosa of the nasopharynx to the brainstem to access the caudal CNS and the spinal cord.

(3) *Systemic Route*

From the nasal cavity, the microbiota can penetrate the rich vascular network in the nasal mucosa, traveling to the brain through the BBB with the systemic pathway. The microbes, like a Trojan-horse, cross the BBB either transcellularly or paracellularly or with the help of infected phagocytes. Then, the presence of these elements alters the BBB function causing increased permeability, encephalopathy, or pleocytosis [4].

### 4.2. Multiple Sclerosis and Nasal Microbiota: Hypothesis concerning a Possible Relationship

From a peripheral point of view, Zhao et al. identified that the composition of nasal microbiota impacts on the onset and recurrence of chronic rhino-sinusitis [33]. This could probably be related to the increase of local inflammation in case of “bad nasal flora” [7]. In fact, it has been proposed that whether (or not) the “good” flora is protective, the “bad” one results in exposure to infection and rino-sinusal inflammation [7,34].

The review of the literature, despite few studies performed on this particular disease (Table 1), identified a higher prevalence of chronic sinusitis (CS) in MS patients compared to healthy individuals [21,22]. CS can be caused both by the change of nasal microbiota and by the altered local immune response [35].Moreover, as well as in allergic rhinitis [36], mastocytes hyperreactivity, which affects MS patients [37], may cause a chronic inflammation of the nasal mucosa, exposing these patients to higher risks of developing rhinological diseases. Finally, because the inflammation changes the nasal pH [38], this change could alter the normal nasal flora with a prevalence of bacteria and viruses that promote inflammation creating a perpetual inflammatory cycle [7].

MS and chronic rhinosinusitis share important clinical and epidemiological similarities. At first, they are chronic inflammations characterized by exacerbation and remission phases associated with minor upper respiratory tract viral infections. Secondly, MS and sinusitis present identical age-of-attack curves, with female prevalence (2:1 women-men). Moreover, women are also affected by the disease slightly earlier than men in both conditions. Finally, the incidence of MS and sinusitis increases with increasing latitude, probably related to climatic factors [19].

At the present time, only a few studies have been published investigating the role played by nasal microbiota dysbiosis in affecting MS development (Table 1). Interestingly, these preliminary clinical and scientific observations support the hypothesis that nasal chronic pathology of different entity and severity can affect MS patients. 

Loosen et al. [20] showed that mononucleosis infection is associated with an increased incidence of MS, especially in younger individuals. These data support the evidence of a clear involvement of EBV in the pathophysiology of MS. The pathological trigger could be related to reprogramming of latently infected B lymphocytes and the chronic presentation of viral antigens. These conditions cause as autoreactivity through molecular mimicry.

Because neuro-inflammation prevails neuro-degeneration, especially in the early phases of MS, the change of nasal microbiota flora as a consequence of chronic rino-sinusal disorders might indirectly affect neuro-inflammation. Preponderance of “bad” nasal flora; which increases inflammation in the nose instead of limiting this phenomenon, can also worsening the nasal environment; this change could increase the risk of suffering from viral infection [7]. Some viruses, such as COVID-19, might expose to high risk of relapsing event in MS [39], because they can raise the concentration of pro-inflammatory elements. We speculate that SARS-CoV-2 might cause molecular mimicry like EBV; however, only long-term observational studies could confirm this hypothesis. As of today, we only know that the severity of COVID-19 disease could be related to the good composition of nasal flora [39,40].

Additionally, even the products of bad nasal microbiota can have a negative effect on the relapsing and progression of MS. These products could reach the brain, increasing the inflammation and the number of relapses, exactly as observed in the case of gut flora in PD [2,3,4]. The inflammation caused by these bad microbiomes can spread from the nose to the brain impacting on the onset of MS in predisposed patients and on relapses [41,42].

Nasal microbiota composition could change even for the treatment used for CS, i.e., nasal spray steroids or antibiotic that have a negative impact on the nasal flora. Moreover, mastocytes in the nose of MS patients has supposed to be hyperreactive, this could increase of local inflammation and change the acidification of the environment; these local events tend to promote bacteria (bad/inflammatory) that may reactivate CS. 

As additional, in acute phase CS is treated with drugs that trigger a negative cycle, as previously underlined. The modified nasal environment thus exposes these patients to recurrent infections, higher risk of contracting upper respiratory viral infection and, perhaps, increases the risk of neuro-inflammation.

Specific studies analyzing the nasal microbiota and its metabolism in patients affected by neuro-inflammatory disorders, such as MS, should be performed; in fact, treatments able to change the nasal flora could be beneficial both to fight the rhino-sinusal infections and might reduce the risk of recurrent episode of neuro-inflammation. 

In the near future, the nasal microbiota could be an ally in preventing neuro-degenerative disease by reducing neuro-inflammatory events.

## Figures and Tables

**Table 1:** Studies investigating the possible relationship between MS and nasal microbiota dysregulation.

Authors	Observational Model	Number of Cases Included in Each Study	Aim	Interventions	Results
Gay et al. [19]	MS human autopsy tissues	21	(1) To identify bacterial toxins or antigens in MS autopsy tissues (2) To search for specific bacterial antibody in the CSF of MS cases	(1)Ttissues were screened for bacterial antigens using immunohistological methods (2) Oligoclonal IgG in CSF were screened using isoelectric focusing and antigen imprinting methods	Anti-staphylococcal antibodies detected antigen co-locating with IgG/C3d immune complexes in pre-demyelinatin and in primary lesions
Branton et al. [20]	MS human autopsy tissues and controls	44	To investigate the composition of microbiota in autopsied brain samples from patients with MS and controls nonMS	(1) RNAseq analyses (2) Histopathology, immunohistochemistry and in situ hybridization	(1) RNAseq analyses showed a predominance of Proteobacteria in progressive MS patients’ white matter, associated with increased inflammatory gene expression; (2) Bacterial peptidoglycan immunodetection was correlated with demyelination and neuroinflammation in MS brains
Ergene et al. [21]	Patients with new onset acute optic neuritis and control patients	71	To evaluate paranasal sinus inflammatory changes	Radiological study with MRI	Frequency of the maxillary sinusitis was significantly higher (*p* = 0.02) in patients with optic neuritis than in controls
Jones et al. [22]	Patients affected by MS	108	To evaluate paranasal sinus inflammatory changes	Radiological study with MRI	The incidence of sinus disease is higher than in some other studies of normal population

MS: multiple sclerosis; CSF: cerebral spinal fluid; MRI: magnetic resonance imaging.

## Data Availability

Not applicable.
